# Bacterium Lacking a Known Gene for Retinal Biosynthesis Constructs Functional Rhodopsins

**DOI:** 10.1264/jsme2.ME20085

**Published:** 2020-12-05

**Authors:** Yu Nakajima, Keiichi Kojima, Yuichiro Kashiyama, Satoko Doi, Ryosuke Nakai, Yuki Sudo, Kazuhiro Kogure, Susumu Yoshizawa

**Affiliations:** 1 Microbial and Genetic Resources Research Group, Bioproduction Research Institute, National Institute of Advanced Industrial Science and Technology (AIST), Ibaraki, 305–8566, Japan; 2 Atmosphere and Ocean Research Institute (AORI), The University of Tokyo, Chiba, 277–8564, Japan; 3 Graduate School of Medicine, Dentistry and Pharmaceutical Sciences, Okayama University, Okayama, 700–8530, Japan; 4 Graduate School of Engineering, Fukui University of Technology, Fukui, 910–8505, Japan; 5 Microbial Ecology and Technology Research Group, Bioproduction Research Institute, National Institute of Advanced Industrial Science and Technology (AIST), Hokkaido, 062–8517, Japan

**Keywords:** phylum *Actinobacteria*, rhodopsin, retinal biosynthesis

## Abstract

Microbial rhodopsins, comprising a protein moiety (rhodopsin apoprotein) bound to the light-absorbing chromophore retinal, function as ion pumps, ion channels, or light sensors. However, recent genomic and metagenomic surveys showed that some rhodopsin-possessing prokaryotes lack the known genes for retinal biosynthesis. Since rhodopsin apoproteins cannot absorb light energy, rhodopsins produced by prokaryotic strains lacking genes for retinal biosynthesis are hypothesized to be non-functional in cells. In the present study, we investigated whether *Aurantimicrobium minutum* KNC^T^, which is widely distributed in terrestrial environments and lacks any previously identified retinal biosynthesis genes, possesses functional rhodopsin. We initially measured ion transport activity in cultured cells. A light-induced pH change in a cell suspension of rhodopsin-possessing bacteria was detected in the absence of exogenous retinal. Furthermore, spectroscopic analyses of the cell lysate and HPLC-MS/MS analyses revealed that this strain contained an endogenous retinal. These results confirmed that *A. minutum* KNC^T^ possesses functional rhodopsin and, hence, produces retinal via an unknown biosynthetic pathway. These results suggest that rhodopsin-possessing prokaryotes lacking known retinal biosynthesis genes also have functional rhodopsins.

Microbial rhodopsins are seven-transmembrane proteins containing a protein moiety (apoprotein) and the light-absorbing chromophore, retinal. The first member of this family of photoreceptor proteins, bacteriorhodopsin, was discovered in halophilic archaea and functions as a light-driven outward H^+^ pump (Oesterhelt and Stoeckenius, 1971). Microbial rhodopsin genes are widely distributed, not only among prokaryotes, but also among fungi (Brown, 2004), eukaryotic algae (Marchetti *et al.*, 2012), and giant viruses (Yutin and Koonin, 2012). Upon illumination, the isomerization of retinal causes a conformational change in rhodopsin, and microbial rhodopsin functions as an ion pump (Oesterhelt and Stoeckenius, 1971), ion channel (Nagel *et al.*, 2002), or light sensor (Spudich, 1998). All microbial rhodopsins are activated via light absorption by retinal, which binds to a lysine in the middle of the seventh transmembrane helix (Lys216; residue number in bacteriorhodopsin). The opsin apoprotein itself cannot receive light without a chromophore and is, thus, considered to be non-functional.

Despite the importance of retinal for functionalizing rhodopsin, the biosynthesis of this chromophore was not fully understood until 2005. A putative gene involved in the retinal biosynthetic pathway was initially discovered in the halophilic archaea, *Halobacterium halobium*; this gene influenced rhodopsin gene expression and was named the bacterio-opsin-related protein (*brp*) (Betlach *et al.*, 1984). A bacterio-opsin-related protein homolog (*blh*) gene was also reported in *Halobacterium* sp. NRC-1 (now recognized as *Halobacterium salinarum* NRC-1) (Ng *et al.*, 2000). *H. salinarum* NRC-1 has both *brp* and *blh* in its genome; nevertheless, the roles of these proteins remained unknown, similar to the biosynthetic pathway of retinal. In 2001, Peck *et al.* showed that *brp* and/or *blh* genes are required for functionalizing rhodopsin (Peck *et al.*, 2001), although these proteins were still suggested to play a role in the transport of retinal or its binding to rhodopsin in cells, rather than in its biosynthesis. In rhodopsin-possessing prokaryotes, the retinal biosynthetic pathway was identified within the same operon as the rhodopsin gene (Sabehi *et al.*, 2005). Sabehi *et al.* confirmed that the *blh* gene encoded a β-carotene 15,15′ dioxygenase, which cleaves β-carotene to retinal. Therefore, *brp* and *blh* were presumed to be homologs, both encoding β-carotene 15,15′ dioxygenase. Sequence homology between the *brp* and *blh* genes is so high that they cannot be clearly distinguished from each other (Nakajima *et al.*, 2018). In contrast, in a cyanobacteria-specific pathway, retinal is produced by a 15,15′-monooxygenase encoded by the *diox1* gene (Ruch *et al.*, 2005). This monooxygenase was also shown to oxidatively cleave β-apo-carotenals in an asymmetric manner, forming retinal. Based on previous findings, rhodopsin-possessing prokaryotes are assumed to produce retinal and, thus, functional rhodopsin.

Despite these earlier theories, recent genomic and metagenomic surveys showed that some prokaryotes that possess rhodopsin genes lack a *blh* homolog (Dupont *et al.*, 2012; Keffer *et al.*, 2015; Denef *et al.*, 2016; Pinhassi *et al.*, 2016; Thiel *et al.*, 2017: Table S1). Organisms such as SAR86, Marine Group II, and CL500-11 are considered to possess rhodopsin genes and are abundant in certain aquatic environments (Morris *et al.*, 2002; Iverson *et al.*, 2012; Okazaki *et al.*, 2012); therefore, these rhodopsin-possessing yet *blh*-lacking prokaryotes appear to be common in the natural environment. A physiological study reported that the rhodopsin-possessing Actinobacteria, *Rhodoluna lacicola* MWH-Ta8^T^ (Hahn *et al.*, 2014), which lacks the *blh* gene, did not exhibit ion-transporting activity until its culture medium was supplied with exogenous retinal (Keffer *et al.*, 2015). Therefore, the rhodopsin present in *R. lacicola* was considered to be non-functional and the strain appeared to obtain retinal from the surrounding environment. However, it currently remains unclear whether all prokaryotes that lack *blh* and *diox1* are unable to endogenously produce retinal.

We herein conducted a functional analysis of a rhodopsin and chemical analyses of pigments from the cosmopolitan freshwater Actinobacterium isolate, *Aurantimicrobium minutum* KNC^T^ (Nakai *et al.*, 2015), which belongs to the same family as *R. lacicola*. The *A. minutum* genome was reported in 2016 (Nakai *et al.*, 2016); it contains a gene that encodes a putative light-driven, proton-pumping rhodopsin, Xanthorhodopsin (Balashov *et al.*, 2005) -like rhodopsin (XLR), but lacks the *blh* gene. We measured XLR proton-pumping activity and performed a spectroscopic analysis and high-performance liquid chromatography-tandem mass spectrometry (HPLC-MS/MS) to clarify whether *A. minutum* produces retinal for the construction of a functional rhodopsin in the absence of the *blh* gene.

## Materials and Methods

### Strain and growth conditions for *A. minutum* KNC^T^

*A. minutum* KNC^T^ was originally isolated from a river in Japan (Nakai *et al.*, 2015). The strain used in the present study was provided by the Biological Resource Center, NITE (NBRC) (Chiba, Japan). *A. minutum* cells were incubated in 300‍ ‍mL of medium (1‍ ‍g‍ ‍L^–1^ Bacto Nutrient Broth, 1‍ ‍g L^–1^ Bacto Tryptone, and 1‍ ‍g‍ ‍L^–1^ Bacto Yeast extract [BD Bioscience]) modified from NSY medium (Hahn *et al.*, 2003) under light (approximately 35‍ ‍μmol photons s^–1^‍ ‍m^–2^) or dark conditions at 25°C for 20 days. Cell growth was measured based on assessments of optical density at 660 nm (OD_660_) every 2 days using a spectrophotometer (Digital Colorimeter AC-114; Optima). After an incubation for 10 days, cells were centrifuged in preparation for the measurement of XLR proton-pumping activity and subsequent analyses.

### Measurement of light-driven proton-pumping activity

Light-driven proton-pumping activity was measured according to the method described by Yoshizawa *et al.* (2012), with the following modifications. Cells in approximately 120–160‍ ‍mL of modified NSY medium were collected by centrifugation at 7,000×*g* at 4°C for 8‍ ‍min. Cells were then washed three times with 100‍ ‍mM NaCl and resuspended in 6‍ ‍mL of 100‍ ‍mM NaCl. The light source was a 300-W xenon lamp (MAX-303; Asahi Spectra), and 6‍ ‍mL of the cell suspension was initially placed in the dark and then irradiated for 5‍ ‍min through 450-±10-nm, 520-±10-nm, and 580-±10-nm bandpass filters (MX0450, MX0520, and MX0580; Asahi Spectra). The light intensity of each wavelength was fixed at approximately 7 mW cm^–2^ using an optical power meter (#3664; Hioki) with an optical sensor (#9742; Hioki). pH was measured using a pH meter (LAQUA F-72; Horiba). Proton-pumping activity was defined as the slope in the 30‍ ‍s following the onset of illumination. This initial slope was normalized to that obtained prior to illumination. The first measurement was performed without external retinal, and the second measurement was conducted after the addition of retinal dissolved in 100% ethanol (at a final concentration of 20‍ ‍μM). All measurements were performed at 4°C.

### Gene preparation and ion transport measurements of XLR in *Escherichia coli* cells

The *A. minutum* DNA fragment encoding XLR (named AmXLR; WP_096383469.1) was chemically synthesized with codon optimization for *E. coli* by Eurofins Genomics. This gene fragment was inserted into the *Nde*I and *Xho*I sites of the pET21a vector (Novagene); consequently, the plasmid encoded AmXLR with hexahistidine at the C terminus. The plasmid was transformed into *E. coli* strain C41 (DE3) (Lucigen). *E. coli* cells with the plasmid were initially incubated at 37°C on an LB-medium agar plate supplemented with ampicillin (final concentration, 100‍ ‍μg mL^–1^), and were then incubated at 37°C in 100‍ ‍mL of 2×YT medium supplemented with ampicillin (final concentration, 100‍ ‍μg mL^–1^) as a pre-culture. After pre-culturing, cells were incubated at 37°C in 100‍ ‍mL of modified NSY medium, which was also used for the incubation of *A. minutum* KNC^T^. Protein expression was induced by adding 0.2‍ ‍mM isopropyl β-D-1-thiogalactopyranoside (IPTG) when OD_660_ reached approximately 0.4. All-*trans* retinal (Sigma-Aldrich) was added to one flask (final concentration 10‍ ‍μM; cell-retinal [+]) and not to the other flask (cell-retinal [–]). Rhodopsin-expressing cells were collected by centrifugation (4,200×*g* for 3‍ ‍min), washed three times, and then re-suspended in 100‍ ‍mM NaCl. A 300-W MAX-303 xenon lamp was used as the light source, and 6‍ ‍mL of the cell suspension was initially placed in the dark and then irradiated through the 520-±10-nm bandpass filter for 5‍ ‍min. pH was monitored using a LAQUA F-72 pH meter. The experiment was repeated using cells incubated with retinal under the same conditions after the addition of the protonophore CCCP (final concentration, 30‍ ‍μM) to inhibit proton transport. All measurements were performed at 4°C.

### Spectroscopic analysis of XLR in *A. minutum* cells

The *A. minutum* cells used for the spectroscopic analysis were incubated under the same conditions as those outlined above for the measurement of proton-pumping activity. Approximately 300‍ ‍mL of modified NSY medium was concentrated by centrifugation at 7,000×*g* at 4°C for 8‍ ‍min. Half of the resulting pellet was suspended in buffer containing 50‍ ‍mM Tris-HCl and 0.1 M NaCl (pH 7.0) and then ultrasonicated for 80 cycles of 1‍ ‍min on and 1‍ ‍min off (UD-211; TOMY Seiko). The supernatant was then obtained by centrifugation at 35,600×*g* at 4°C for 10‍ ‍min, followed by ultra-centrifugation at 178,000×*g* at 4°C for 30‍ ‍min (himac CP56G; Hitachi Koki, P50A2 rotor; Hitachi Koki) to separate the pellet containing the membrane fraction. The pellet was homogenized on ice and suspended in Tris-HCl buffer at pH 7.0. Hydroxylamine was added to the suspension to a final concentration of 100‍ ‍mM, and absorption spectra were measured every 10‍ ‍min using the ultraviolet-visible spectrophotometer UV-2450 with the ISR2200 integrating sphere (Shimadzu). The reaction constant of the reaction, τ, was estimated from a single exponential fit:

y=y0+A*exp(–x/τ)

where y and x are the absorption change and reaction time, respectively.

### HPLC-MS/MC analyses

*A. minutum* cells used in the HPLC-MS/MS analysis were incubated under the same conditions as those described above. Cells in approximately 300‍ ‍mL of culture medium were pelleted by centrifugation at 7,000×*g* at 4°C for 8‍ ‍min, and the supernatant was completely removed. In the HPLC-MS/MS analysis, aliquots of acetone were added to the frozen pellet in a microtube, which was then placed in an ice-cooled ultrasonication bath for pigment extraction. Samples were then ultrasonically homogenized for a few minutes, and acetone supernatants were immediately separated from the particulate material by centrifugation and directly injected into the HPLC apparatus for analysis.

The HPLC-MS/MS instrument consisted of a Shimadzu Nexera X2 liquid chromatography system, comprising a CBM-20A communication bus module, two DGU-20A3R/5R HPLC degassing units, three LC-30AD solvent delivery units constituting a ternary pumping system, an SIL-30AC autosampler, CTO-20AC column oven, and LCMS-8030 triple quadrupole mass spectrometer connected through an atmospheric pressure chemical ionization (APCI) interface (Shimadzu). The system was then coupled to a personal computer configured to run Shimadzu LabSolution software. Reverse-phase HPLC was performed under the following conditions: column, Zorbax Eclipse Plus C18 (Rapid Resolution HT, 3.0×100‍ ‍mm, 1.8-μm silica particle size; Agilent Technologies); eluent, the ternary gradient program summarized in Table S2; and flow rate, 0.5‍ ‍mL min^–1^. All mobile phases were degassed *in vacuo* using ultrasonication. Mobile-phase reservoir bottles were designed to prevent any contact between the mobile phases and air during the analysis. The solvents used for the HPLC mobile phases included LC/MS-grade distilled water and methanol purchased from Kanto Chemical and LC/MS-grade formic acid and HPLC-grade acetone purchased from Waco Pure Chemical Industries. APCI was set to the following conditions: nebulizer gas flow, 2.2‍ ‍L‍ ‍min^–1^; interface temperature, 320°C; desolvation line temperature, 140°C; heat-block temperature, 250°C; drying gas flow, 3‍ ‍L‍ ‍min^–1^. The parameters of the Q1 scan in the positive ion mode of the mass spectrometer were set as follows: scan range, *m/z* 100.00–400.00; event time, 0.200. Parameters for the detection of retinal during multiple reaction monitoring (MRM) in the positive ion mode were as follows: precursor ion ([M+H]^+^), *m/z* 285.25; product ion, *m/z* 160.10; dwell time, 10 ms; collision energy (CE), –10 V.

## Results and Discussion

### XLR proton-pumping activities of *A. minutum* with and without external retinal

*A. minutum* cells were incubated for 20 days under light or dark conditions. No significant differences were observed in the growth of *A. minutum* between the light and dark conditions until the mid-log phase (Fig. S1). Maresca *et al.* (2019) reported that *Aurantimicrobium* spp. grow more rapidly in the light than in the dark. The discrepancy between the present results from their findings may be due to differences in the medium components, the strain phenotype, or both. *A. minutum* cells incubated for 10 days were used to measure light-driven proton-pumping activities because the maximum OD_660_ during the incubation period was observed at 10 days; no significant differences were observed in OD_660_ between the light and dark conditions.

**Fig. 1.**
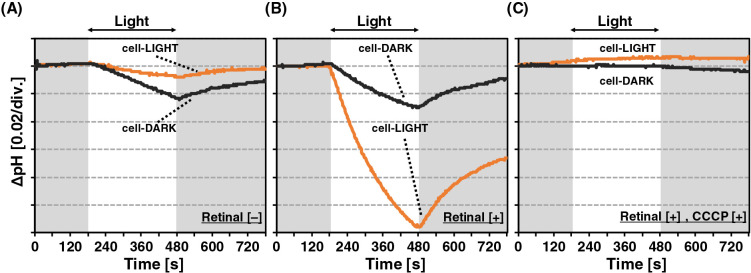
Light-induced pH changes in *Aurantimicrobium minutum* cells in suspension. (A, B) Measurement without (A) and with (B) an external supply of retinal. (C) After the measurement with the addition of retinal, the protonophore CCCP was added and the measurement was repeated. Orange and black lines indicate cell-LIGHT and cell-DARK, respectively. The cell suspension was illuminated with green light (520‍ ‍nm) for 300‍ ‍s, and temperature was maintained at 4°C. div, division.

Light-induced pH changes in suspensions of *A. minutum* cells were measured using cells incubated in the light (cell-LIGHT) or dark (cell-DARK). A light-induced pH change was observed in both cell-LIGHT and cell-DARK samples in the absence of exogenous retinal (Fig. 1A) despite the absence of *blh* in the genome of *A. minutum*. To exclude the possibility that the chromophore was supplied by modified NSY medium, we measured proton-pumping activities using *E. coli* cells that heterologously expressed AmXLR, and a light-induced pH change was not detected without retinal (Fig. S2; gray line “Retinal [–]”). In contrast, a light-induced pH change was detected in AmXLR-expressing *E. coli* cells when retinal was added to the medium (Fig. S2; orange line “Retinal [+]”). These results suggest the following: (1) AmXLR expressed in *E. coli* functions as a light-driven proton pump if retinal is externally supplied, (2) *E. coli* does not produce retinal, and (3) modified NSY medium did not contain retinal. Therefore, *A. minutum* constructs a functional rhodopsin, the XLR apoprotein of which binds to an endogenous chromophore.

When retinal was externally supplied, a marked decrease in pH was induced by light in cell-LIGHT (Fig. 1B; orange line), whereas a markedly smaller reduction was observed in pH in cell-DARK (Fig. 1B; black line). The addition of the protonophore, CCCP, to the same cells abolished light-induced pH changes (Fig. 1C; orange and black lines). Furthermore, the light-induced pH change at green light (520‍ ‍nm) was larger than those at orange (580‍ ‍nm) and blue (450‍ ‍nm) lights (Fig. S3). In other words, proton-pumping activity was the strongest at a wavelength close to the absorption maximum of rhodopsin. These results showed that pH changes were due to the light-driven proton pumping of XLR in *A. minutum*. In the analysis of XLR proton-pumping activity in cells incubated for 10 days, the initial slope of cell-LIGHT with an external supply of retinal was significantly higher than that of cell-DARK with an external supply of retinal (Fig. S4). Based on equal photon flux, proton-pumping activity was presumably proportional to the number of rhodopsin molecules activated by the binding of externally supplied retinal. Each rhodopsin molecule contains only a single retinal molecule; therefore, the quantity of retinal supplied to the cell suspension (20‍ ‍mM) was excessive, and the increase observed in proton-pumping activity must have reflected the quantity of the XLR apoprotein expressed prior to the addition of retinal. Therefore, these results indicated that more XLR was expressed under light conditions than under dark conditions. Nevertheless, it is important to note that a significant difference remained between the proton-pumping activity in cell-LIGHT with and without externally supplied retinal (Fig. S4). These results suggested that some, but not all, XLR apoproteins expressed in cell-LIGHT were functionalized by accepting some chromophores that must have been endogenously supplied by *A. minutum*. In other words, *A. minutum* may produce a smaller amount of the chromophore than the XLR apoprotein, such that the amount of functional XLR in *A. minutum* is limited by the concentration of the endogenous chromophore, possibly retinal, and some of the XLR expressed is non-functional. In the rhodopsin-possessing bacterium, *Vibrio* sp. AND4, light-enhanced long-term survival during starvation was suggested to have been mediated by proton-pumping rhodopsin (Gómez-Consarnau *et al.*, 2010; Akram *et al.*, 2013). Proton-pumping rhodopsin may generate ATP under irradiation with light and reduce the consumption of organic matter and/or oxygen in the medium used for energy metabolism. Once the bacterium maintains the same metabolic activity, the conservation of organic matter via this rhodopsin function is considered to prolong the survival of the bacterium (Steindler *et al.*, 2011). The production of the chromophore by *A. minutum* may be up-regulated under these conditions, such that the utilization of rhodopsin becomes more important for its survival. Therefore, similar to other *blh*-lacking prokaryotes, native *A. minutum* may utilize retinal externally supplied by other retinal producers inhabiting the same environment if it becomes available.

### Spectroscopic analysis of native *A. minutum* cells after hydroxylamine bleaching

**Fig. 2.**
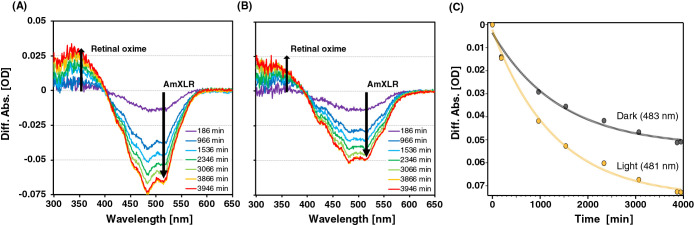
Different spectra of the lysate at various times after the addition of hydroxylamine relative to no hydroxylamine. (A) Sample from cell-LIGHT. (B) Sample from cell-DARK. Each line indicates a difference spectrum at the representative times after the addition of hydroxylamine. Arrows indicate an increase in the absorbance of retinal oxime or decrease in the absorbance of AmXLR. (C) Estimation of the time constant of the reaction (τ) on cell-LIGHT and cell-DARK by a single exponential fitting.

We performed a spectroscopic analysis to examine whether *A. minutum* produces retinal as an endogenous chromophore of XLR. The spectra from cell-LIGHT and cell-DARK were obtained from the lysate after the addition of hydroxylamine. In the presence of retinal-bound rhodopsin, an absorbance peak of retinal oxime was expected to emerge upon the bleaching of rhodopsin (Sudo *et al.*, 2002). As shown in Fig. 2, the absorbance intensity at the AmXLR absorption maximum (approximately 532 nm) decreased, whereas that at the retinal oxime maximum (approximately 363 nm) increased. Furthermore, τ of cell-LIGHT (τ-light) was estimated at 481-nm datasets, while τ of cell-DARK (τ-dark) was estimated at 483-nm datasets because these wavelengths are the peak wavelengths of the different absorptions (Fig. 2C). τ-light and τ-dark were approximately 1,300‍ ‍min (22 h) and 1,400‍ ‍min (23 h), respectively. Since the time constant of the reaction of BR was estimated to be approximately 10 h (Subramaniam *et al.*, 1991), τ-light and τ-dark were not markedly different from τ of BR.

These results suggest that rhodopsin underwent discoloration by desorbing retinal oxime, indicating that at least some XLR in *A. minutum* KNC^T^ bound retinal. However, these differences in spectra may have resulted from a chromophore with a formyl group that is analogous to retinal. Therefore, we performed HPLC-MS/MS analyses to establish whether the chromophore produced by *A. minutum* is retinal.

### Identification of endogenous retinal by the HPLC-MS/MS analysis

**Fig. 3.**
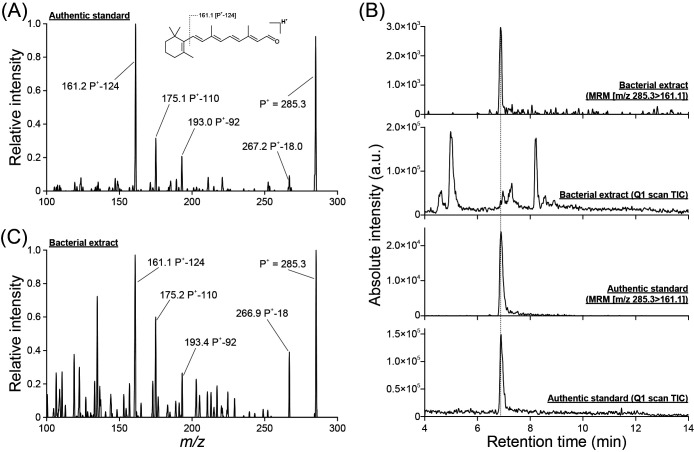
LC-MS/MS spectra from an authentic standard and bacterial extract. (A) Product ion spectrum of the authentic standard all-*trans* retinal on a precursor ion set with *m/z*=285.3. (B) Total ion chromatogram (TIC) and multiple reaction monitoring (MRM) of a bacterial extract and authentic standard. The dotted line indicates the retention time of all-*trans* retinal. (C) Product ion spectrum of the MRM peak of the bacterial extract on a precursor ion set with *m/z*=285.3.

We obtained the product ion spectrum of the authentic standard of all-*trans* retinal on the expected precursor mass [M+H]^+^ with *m/z*=285.3, identifying the primary ion product with *m/z*=161.2, which was consistent with the theoretical value *m/z*=161.1 (Fig. 3A). Unique peaks with identical retention times were observed in the mass spectra on the expected MRM transition of retinal [*m/z*=285.3>*m/z*=161.1] of both the authentic standard and *A. minutum* extract (Fig. 3B), suggesting the occurrence of retinal in *A. minutum* KNC^T^. Furthermore, the fragmentation pattern of the product ion spectrum on the expected precursor mass was obtained at the identified peak of the *A. minutum* extract (Fig. 3C) and matched well with the pattern of the authentic standard (Fig. 3A), confirming the detection of retinal in the bacterial extract. On the other hand, we did not detect any carotenoid signals, which potentially are retinal precursors, under the HPLC-MS/MS conditions used in the present study.

Microbial rhodopsins have several retinal isomers, such as all-*trans*, 13-*cis*, 11-*cis*, and 9-*cis*. Of these, most microbial rhodopsins (particularly proton-pumping rhodopsins) only work with all-*trans* retinal. Therefore, we assumed that AmXLR has an all-*trans* retinal as a chromophore. However, Fig. S4 shows that this strain did not produce sufficient amounts of retinal capable of binding to all XLR apoproteins. Since only two culture conditions (light and dark) were employed in the present study, it currently remains unclear whether *A. minutum* produced sufficient retinal under different culture conditions. Peck *et al.* (2001) showed that the amount of functional rhodopsin (bacteriorhodopsin) increased following the addition of exogenous retinal in *H. salinarum*. This increase was greater in a *brp* gene deletant (that only possessed the *blh* gene), indicating that not all rhodopsin are functional, even though this archaeon produces retinal. In other words, the amount of retinal may be less than that of rhodopsin apoproteins among some rhodopsin-possessing prokaryotes.

### Hypothesis of an unknown retinal biosynthetic pathway

Although we showed that *A. minutum* KNC^T^ produced retinal, the genes responsible for its biosynthesis have not yet been identified. Among rhodopsin-possessing Actinobacteria, a lycopene cyclase encoded by the *crtYc/Yd* genes catalyzes the conversion of lycopene to β-carotene, which is then supplied for endogenous retinal (Dwulit-Smith *et al.*, 2018). However, *A. minutum* has putative *crtYe/Yf* cluster genes, which encode C50 carotenoid epsilon cyclase (Krubasik *et al.*, 2001; Netzer *et al.*, 2010), not *crtYc/Yd* genes. Representative *crtYe/Yf* genes are found in *Corynebacterium glutamicum* (accession numbers: AUI03847 and AUI03848) and *Micrococcus luteus* (accession numbers: AYO50480 and AYO50481), to which the putative genes of *A. minutum* are closely related; however, they are less closely related to the *crtYc/Yd* clusters (Fig. S5). It has not yet been established whether C50 carotenoid epsilon cyclase also converts lycopene (C40 carotenoid) to β-carotene; therefore, *A. minutum* KNC^T^, similar to other rhodopsin-possessing Actinobacteria, may not produce β-carotene from lycopene. In contrast, *A. minutum* has a putative *crtEb* gene encoding lycopene elongase, which converts lycopene to a C50 carotenoid. C50 carotenoid epsilon cyclase encoded by the *crtYe/Yf* genes may form cyclic ends to convert a β-carotene analog of the C50 carotenoid with an extended isoprenyl chain. Since *A. minutum* possesses genes involved in C50 carotenoid biosynthesis, we hypothesized that this strain may have an oxygenase that asymmetrically cleaves cyclic C50 carotenoid to produce all-*trans* retinal (Fig. S6). Furthermore, according to the phylogenetic tree shown in Fig. S5, *R. lacicola* MWH-Ta8^T^, which was not considered to produce retinal, at least possesses the *crtYe/Yf* genes (and also a *crtEb* gene). Therefore, *R. lacicola* may also produce retinal through an unknown pathway similar to that in *A. minutum*. However, the present results do not exclude the possibility of a retinal biosynthesis pathway without the C50 carotenoid as an intermediate. While a β-carotene 15,15′ dioxygenase cleaves one β-carotene to two retinal, asymmetrical cleavage may convert one C50 carotenoid to one retinal, such as 15,15′-monooxygenase, encoded by the *diox1* gene. In that case, the number of retinal molecules produced by asymmetrical cleavage is half the number of retinal molecules produced by dioxygenase cleavage. This may be one of the reasons why *A. minutum* only functionalizes some of the XLR expressed.

In conclusion, we herein report that some XLR rhodopsins in the freshwater Actinobacterium, *A. minutum* KNC^T^, which possesses a rhodopsin gene and lacks the known retinal biosynthetic genes *blh* and *diox1*, are functional. The cleavage of β-carotene is not an abiotic reaction, but an enzymatic reaction that produces retinal (Olson and Hayaishi, 1965); therefore, *A. minutum* KNC^T^ must produce (all-*trans*) retinal using an unknown retinal biosynthetic gene(s) for functionalizing rhodopsin. Although rhodopsins in prokaryotes that lack *blh* or *diox1* were previously considered to be non-functional, the endogenous production of retinal was confirmed in this strain. Interestingly, diverse organisms that include strains belonging to the phyla *Deinococcus-Thermus*, *Firmicutes*, *Proteobacteria*, *Bacteroidetes*, *Chloroflexi*, and *Euryarchaeota* have putative rhodopsin genes, but lack *blh* and *diox1* (data not shown); however, it currently remains unclear whether these organisms endogenously produce retinal without the currently known retinal biosynthetic genes. Further studies on these strains may reveal how widespread retinal production is in *blh*-lacking strains and also how these rhodopsin-possessing prokaryotes obtain retinal to construct functional rhodopsins.

## Citation

Nakajima, Y., Kojima, K., Kashiyama, Y., Doi, S., Nakai, R., Sudo, Y., et al. (2020) Bacterium Lacking a Known Gene for Retinal Biosynthesis Constructs Functional Rhodopsins. *Microbes Environ ***35**: ME20085.

https://doi.org/10.1264/jsme2.ME20085

## Supplementary Material

Supplementary Material
