# Supplementary material for: Bacterium Lacking a Known Gene for Retinal Biosynthesis Constructs Functional Rhodopsins
Source: Microbes Environ. 2020 Dec 5;35(4):ME20085. doi: 10.1264/jsme2.ME20085 (PMC7734400; doi:10.1264/jsme2.ME20085)
Supplement: Supplementary file 1 — Supplementary Material [file 35_20085_s1.pdf]

Supplementary information

Table S1. Rhodopsin-possessing bacteria that lack the *blh* gene

**Name**	**Taxonomic group**	**Reference**
SAR86	*Proteobacteria*	Dupont *et al.*, 2012
Roseiflexus sp. RS-1	*Chloroflexi*	Keffer *et al.*, 2015
Rhodoluna lacicola MWH-Ta8^{T}	*Actinobacteria*	Keffer *et al.*, 2015
Marine Group II	*Euryarchaeota*	Pinhassi *et al.*, 2016
CL500-11	*Chloroflexi*	Denef *et al.*, 2016
Bellilinea sp.	*Chloroflexi*	Thiel *et al.*, 2017
Ca. Roseilinea gracile	*Chloroflexi*	Thiel *et al.*, 2017

Table S2. HPLC gradient programs for the ternary solvent system used in the present study.

Time (min)	Solvent			Note
	A (%)	B (%)	C (%)	
0.0 → 1.0	15	85	0	Actual analysis
1.0 → 9.0	15 → 0	85 → 100	0	
9.0 → 14.0	0	100 → 90	0 → 10	
14.0 → 14.1	0	90 → 50	10 → 50	Flushing
14.1 → 20.0	0	50	50	
20.0 → 20.1	0 → 15	50 → 85	50 → 0	Re-
20.1 → 25.0	15	85	0	

Solvent A, distilled water with 0.1% formic acid (FA); B, methanol with 0.1% FA; C, Acetone.

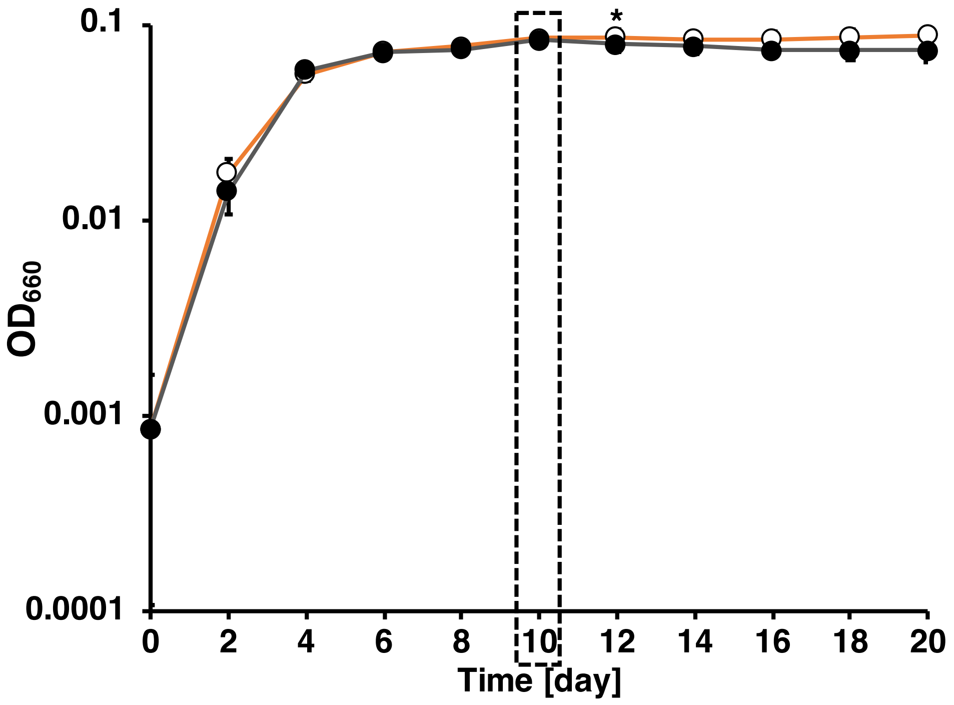

Fig. S1. Growth of *A. minutum* KNC^T. Orange line: cell-LIGHT (white light source, 35 μmol photons s^{-1} m^{-2}); black line: cell-DARK. All cells were incubated at 25 ºC. The asterisk indicates that the mean of the growth under the light condition is significantly different from that under the dark condition with a p-value (=0.017), as calculated with a Student's t test. The dotted line indicates the sampling day for the measurement of proton pumping activity, spectroscopic analysis, and HPLC-MS/MS.

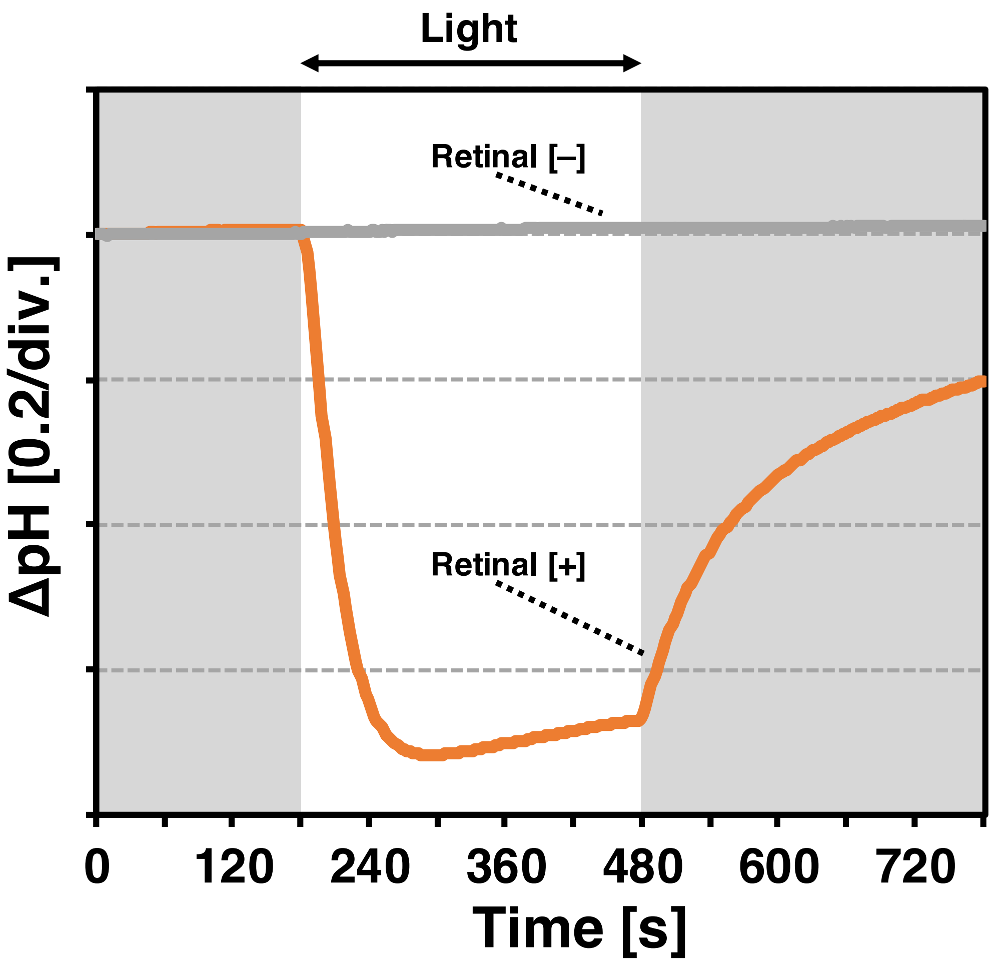

Fig. S2. Light-induced pH changes in AmXLR-expressing *E. coli* cells suspended in 100 mM NaCl. The orange line indicates cells that were incubated with 10 μM all-*trans* retinal. The gray line indicates the cells that were incubated without retinal. Both groups were incubated with 0.2 mM isopropyl β-D-1-thiogalactopyranoside (IPTG) to induce protein expression. The cell suspensions were illuminated with green light (520 nm). Temperature was maintained at 4 °C. div, division.

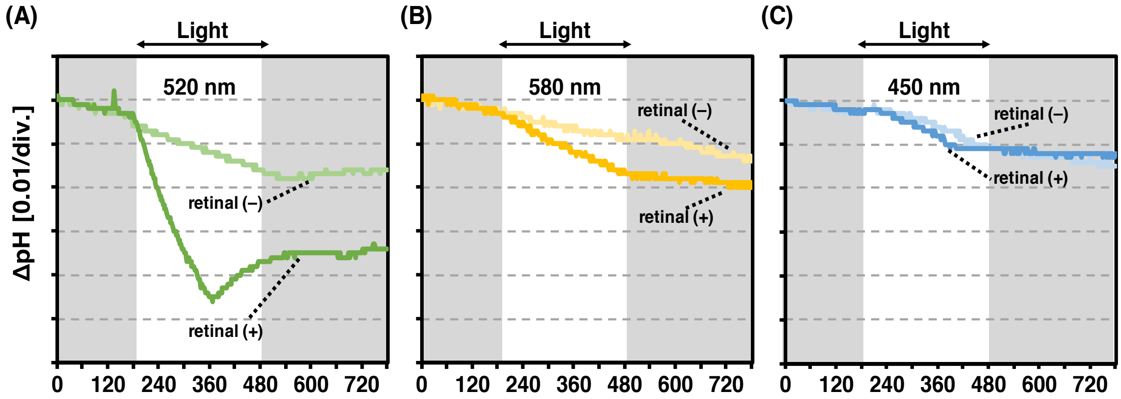

Fig. S3. Light-induced pH changes in *A. minutum* cell suspension at different wavelength lights. The cell-LIGHT sample was used for measurement at green light (520 nm) (A), orange light (580 nm) (B) and blue light (450 nm) (C), respectively. On each wavelength, measurement was performed without retinal (pale line) and with 10 µM all-*trans* retinal (vivid line). Temperature was maintained at 4 °C. div, division.

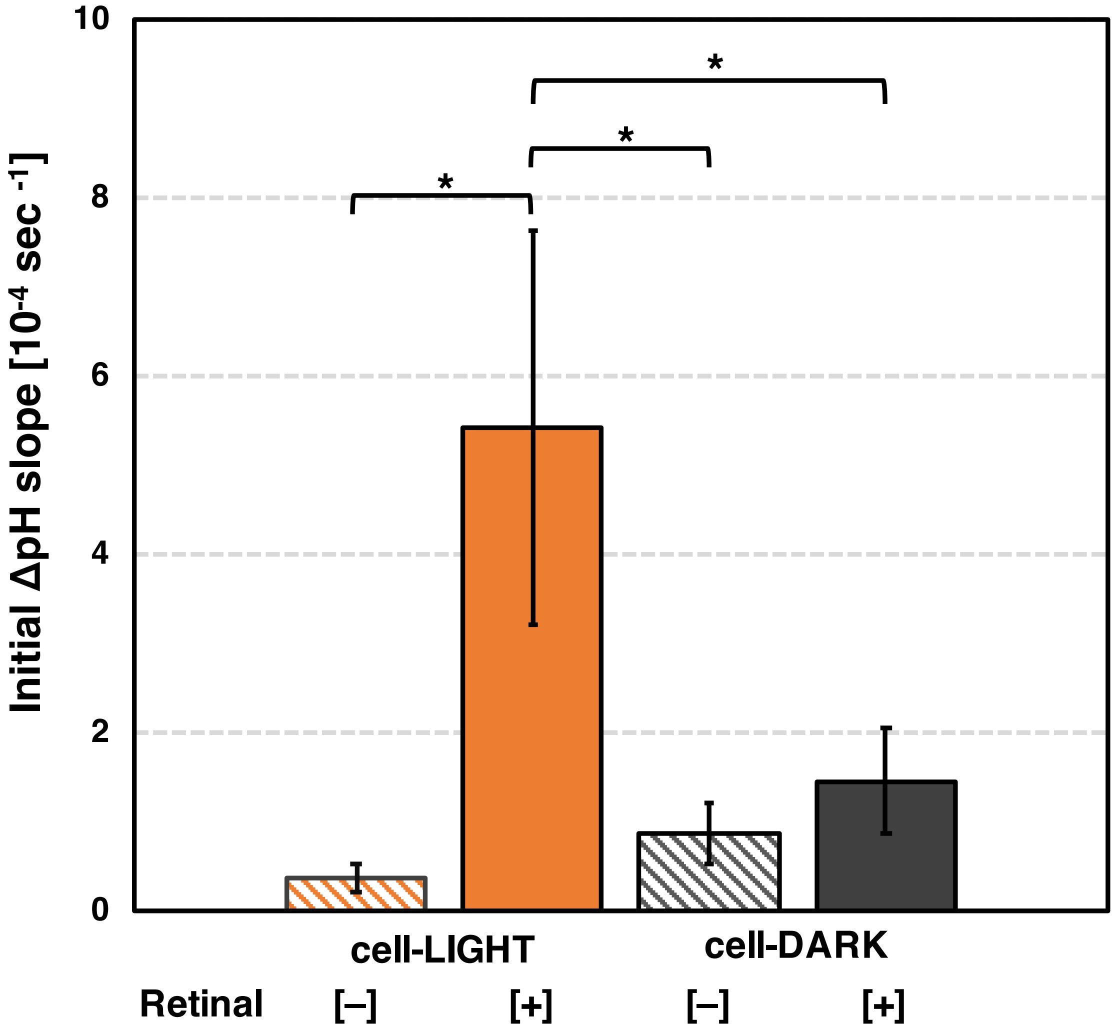

Fig. S4. Proton pumping activity of the XLR in *A. minutum* incubated under light and dark conditions. Retinal [–]: measurement without external supply of retinal; retinal [+]: measurement with external supply of retinal. N = 6 for all measurements. Asterisks indicate that the mean of the proton pumping activity is significantly different with a p-value of < 0.001, as calculated with a Tukey-Kramer test and two-way ANOVA analysis.

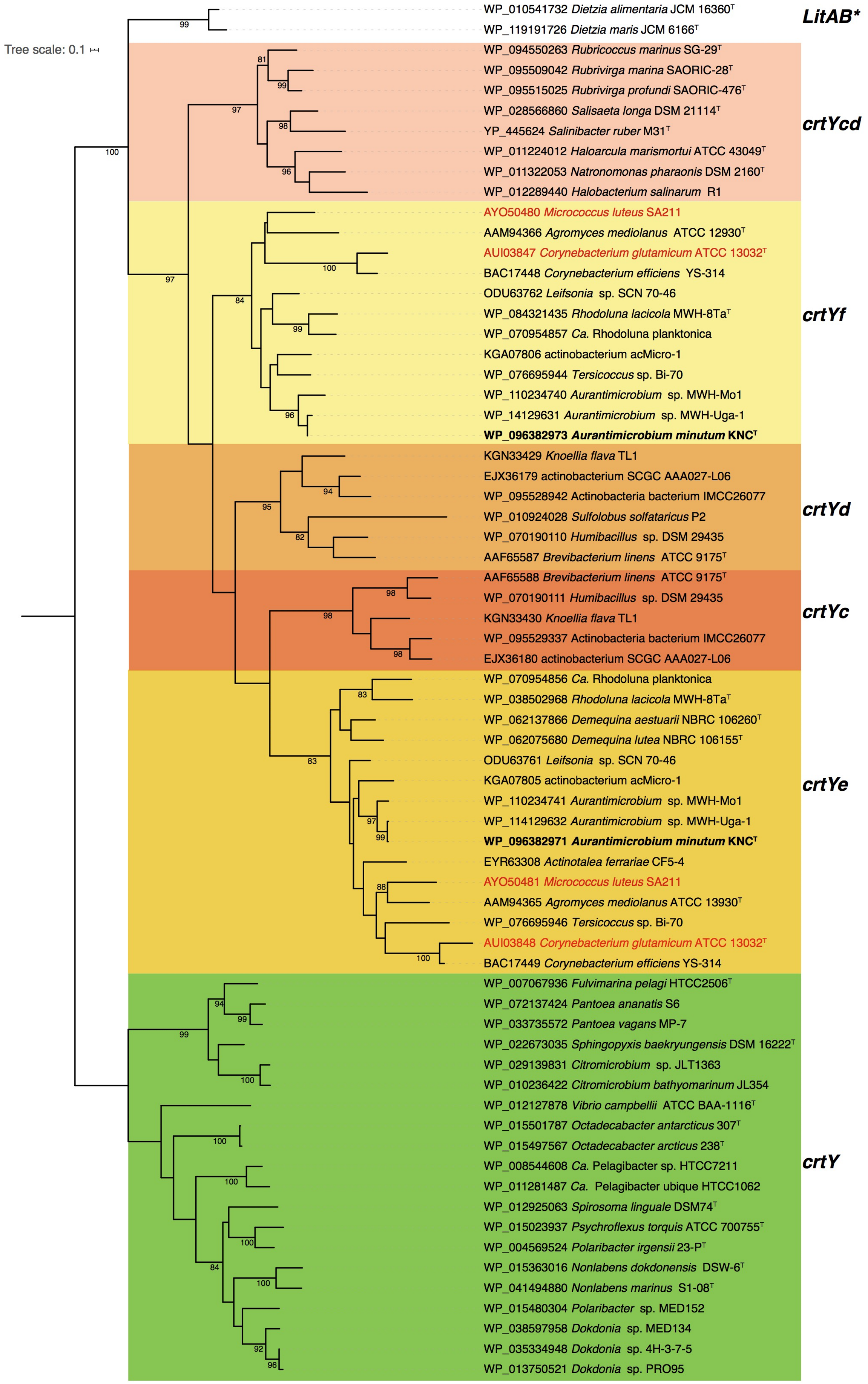
Tree scale: 0.1
LitAB*
WP_010541732 Dietzia alimentaria JCM 16360T
WP_119191726 Dietzia maris JCM 6166T
crtYcd
WP_094550263 Rubricoccus marinus SG-29T
WP_095509042 Rubrivirga marina SAORIC-28T
WP_095515025 Rubrivirga profundi SAORIC-476T
WP_028566860 Salisaeta longa DSM 21114T
YP_445624 Salinibacter ruber M31T
WP_011224012 Haloarcula marismortui ATCC 43049T
WP_011322053 Natronomonas pharaonis DSM 2160T
WP_012289440 Halobacterium salinarum R1
crtYf
AYO50480 Micrococcus luteus SA211
AAM94366 Agromyces mediolanus ATCC 12930T
AUI03847 Corynebacterium glutamicum ATCC 13032T
BAC17448 Corynebacterium efficiens YS-314
ODU63762 Leifsonia sp. SCN 70-46
WP_084321435 Rhodoluna lacicola MWH-8TaT
WP_070954857 Ca. Rhodoluna planktonica
KGA07806 actinobacterium acMicro-1
WP_076695944 Tersicoccus sp. Bi-70
WP_110234740 Aurantimicrobium sp. MWH-Mo1
WP_14129631 Aurantimicrobium sp. MWH-Uga-1
WP_096382973 Aurantimicrobium minutum KNCT
crtYd
KGN33429 Knoellia flava TL1
EJX36179 actinobacterium SCGC AAA027-L06
WP_095528942 Actinobacteria bacterium IMCC26077
WP_010924028 Sulfolobus solfataricus P2
WP_070190110 Humibacillus sp. DSM 29435
AAF65587 Brevibacterium linens ATCC 9175T
crtYc
AAF65588 Brevibacterium linens ATCC 9175T
WP_070190111 Humibacillus sp. DSM 29435
KGN33430 Knoellia flava TL1
WP_095529337 Actinobacteria bacterium IMCC26077
EJX36180 actinobacterium SCGC AAA027-L06
crtYe
WP_070954856 Ca. Rhodoluna planktonica
WP_038502968 Rhodoluna lacicola MWH-8TaT
WP_062137866 Demequina aestuarii NBRC 106260T
WP_062075680 Demequina lutea NBRC 106155T
ODU63761 Leifsonia sp. SCN 70-46
KGA07805 actinobacterium acMicro-1
WP_110234741 Aurantimicrobium sp. MWH-Mo1
WP_114129632 Aurantimicrobium sp. MWH-Uga-1
WP_096382971 Aurantimicrobium minutum KNCT
EYR63308 Actinotalea ferrariae CF5-4
AYO50481 Micrococcus luteus SA211
AAM94365 Agromyces mediolanus ATCC 13930T
WP_076695946 Tersicoccus sp. Bi-70
AUI03848 Corynebacterium glutamicum ATCC 13032T
BAC17449 Corynebacterium efficiens YS-314
crtY
WP_007067936 Fulvimarina pelagi HTCC2506T
WP_072137424 Pantoea ananatis S6
WP_033735572 Pantoea vagans MP-7
WP_022673035 Sphingopyxis baekryungensis DSM 16222T
WP_029139831 Citromicrobium sp. JLT1363
WP_010236422 Citromicrobium bathyomarinum JL354
WP_012127878 Vibrio campbellii ATCC BAA-1116T
WP_015501787 Octadecabacter antarcticus 307T
WP_015497567 Octadecabacter arcticus 238T
WP_008544608 Ca. Pelagibacter sp. HTCC7211
WP_011281487 Ca. Pelagibacter ubique HTCC1062
WP_012925063 Spirosoma linguale DSM74T
WP_015023937 Psychroflexus torquis ATCC 700755T
WP_004569524 Polaribacter irgensii 23-PT
WP_015363016 Nonlabens dokdonensis DSW-6T
WP_041494880 Nonlabens marinus S1-08T
WP_015480304 Polaribacter sp. MED152
WP_038597958 Dokdonia sp. MED134
WP_035334948 Dokdonia sp. 4H-3-7-5
WP_013750521 Dokdonia sp. PRO95

Fig. S5. Maximum-likelihood phylogenetic tree of carotenoid cyclase genes. Amino acid sequences were aligned using MAFFT (Katoh *et al.*, 2002) ver. 7.310, and the tree was estimated using RAxML(ver. 8.2.12) (Stamatakis, 2014) with the following settings: raxmlHPC -f -s input file -p 12345 -x 12345 -# 100 -n output file -m PROTGAMMA. Bootstrap values >80% are indicated as a percentage of the replicates tested. The asterisk (*) indicates genes that belong to the *LitAB* cluster and are not the *crtY* genes but have been identified as genes encoding carotenoid cyclase in a previous study (Klassen, 2010). Red characters indicate representative sequences of *crtYe/Yf* genes.

Klassen, J. L. 2010. Phylogenetic and evolutionary patterns in microbial carotenoid biosynthesis are revealed by comparative genomics. PLoS ONE 5: e11257.2

Katoh, K., Misawa, K., Kuma, K. I., and Miyata, T. 2002. MAFFT: a novel method for rapid multiple sequence alignment based on fast Fourier transform. Nucleic acids research, 30: 3059-3066.

Stamatakis, A. 2014. RAxML version 8: a tool for phylogenetic analysis and post-analysis of large phylogenies. Bioinformatics 30:1312–1313.

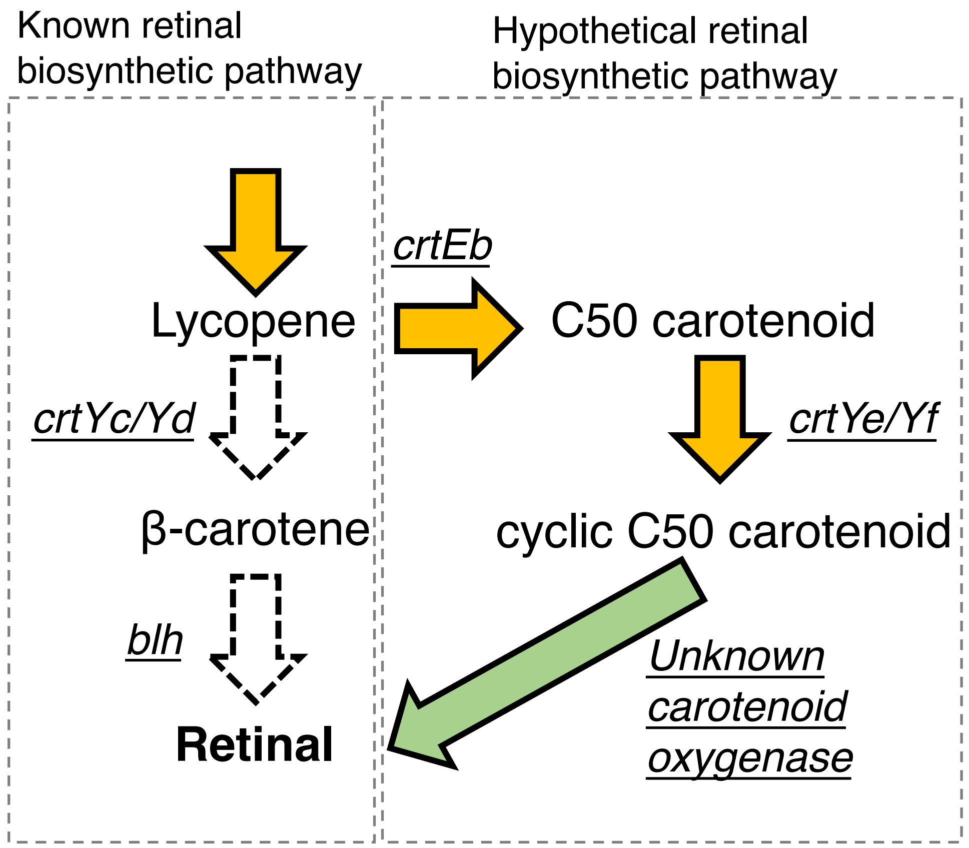

Fig. S6. Known retinal biosynthetic pathway and a hypothetical pathway in *A. minutum* KNC^{T}. Open arrows: genes lacking in *A. minutum*; orange arrow: gene present in *A. minutum*; green arrows: genes required for the pathway hypothesized from this study
